# Feasibility Study of a Novel Magnetic Bone Cement for the Treatment of Bone Metastases

**DOI:** 10.3390/life12091342

**Published:** 2022-08-29

**Authors:** Bowen Ren, Zhenchuan Han, Wenyi Li, Jianheng Liu

**Affiliations:** 1 Department of Orthopedics, The Fourth Medical Centre of Chinese PLA General Hospital, Beijing 100089, China; 2 Chinese PLA Medical School, Beijing 100853, China; 3 Department of Orthopedics, Chinese PLA Rocket Force Characteristic Medical Center, Beijing 100088, China; 4 Department of Orthopedics, Hebei General Hospital, Shijiazhuang 050051, China

**Keywords:** magnetic hyperthermia, magnetite nanoparticles, bone metastases, PMMA, heating efficiency

## Abstract

Bone cement is a crucial material to treat bone metastases defects, and can fill the bone defect and provide mechanical support simultaneously, but the antitumor effect is very limited. Magnetic bone cement not only supports bone metastasis defects but can also achieve magnetic hyperthermia to eliminate tumor cells around the bone defect. However, the physicochemical properties of the bone cement matrix will change if the weight ratio of the magnetic nanoparticles in the cement is too high. We mixed 1 weight percent Zn_0.3_Fe_2.7_O_4_ with good biocompatibility and high heating efficiency into a polymethyl methacrylate matrix to prepare magnetic bone cement, which minimized the affection for physicochemical properties and satisfied the hyperthermia requirement of the alternating magnetic field.

## 1. Introduction

Skeletal-related events are caused by bone metastases, including pain, pathologic fracture, and spinal cord compression, which are the main reasons for the decline of quality of life among advanced oncology patients [1,2]. With advances in medical technology, the survival time of tumor patients has improved. Meanwhile, the number of bone metastases patients has also increased. Previous investigations have reported that there are 300,000 bone metastases patients each year in the USA [3], which may increase in the future.

The spine is the predilection site of bone metastases, and 90% of spinal tumors are metastatic. At present, percutaneous vertebroplasty with bone cement is the main minimally invasive surgery for the treatment of spinal bone metastases. Bone cement is a crucial filling material in the treatment of bone metastases; however, the antitumor effect of conventional bone cement is limited.

The addition of chemotherapy drugs to bone cement struggles to achieve a constant antitumor effect due to the “sudden release effect” [4], and the pharmaceutical activity may also be affected by heat emitted from the polymerization of the cement. Magnetic bone cement, an additional antitumor filling material, induces the antitumor effect by hyperthermia of magnetic materials in the alternating magnetic field (AMF). Because the hyperthermia effect is induced by the relaxation of magnetic nanoparticles in AMF, magnetic bone cement can achieve repeated hyperthermia [5,6,7]. However, physicochemical properties, such as mechanical strength, setting time, and polymerization temperature, will change if the weight percent (wt%) of the magnetic nanoparticles in the bone matrix increases. Previous studies reported that the weight percent of magnetic nanoparticles in magnetic bone cement is approximately 30–60 wt% [5,8,9]. Such a high weight ratio of magnetic nanoparticles not only affects the physicochemical properties but can also increase the cytotoxicity if the bone cement matrix is degradable, such as with calcium phosphate (CPC) and calcium sulfate (CS). Previous investigations have given the reason for this as poor heating efficiency of magnetic nanoparticles [10,11]. Accordingly, improving the heating efficiency of the magnetic nanoparticles will reduce the weight ratio in the bone cement matrix, which will also minimize the effect on physicochemical properties.

In this study, we synthesized 22 nm Zn_0.3_Fe_2.7_O_4_ nanoparticles using the one-pot method, as the nanoparticles have high heating efficiency and good biocompatibility [12]. We then added 1 wt% Zn_0.3_Fe_2.7_O_4_ nanoparticles to polymethyl methacrylate (PMMA) to prepare magnetic bone cement (M-PMMA), which minimizes the affection to the physicochemical properties, and satisfies the hyperthermia requirement in the AMF. The in vitro and in vivo performance of M-PMMA was also evaluated, and 1 wt% Zn_0.3_Fe_2.7_O_4_ PMMA-based bone cement was able to suppress bone metastases and provide a promising clinical transformation.

## 2. Materials and Methods

### 2.1. Preparation of Magnetic PMMA-Zn_0.3_Fe_2.7_O_4_ Bone Cement

Zinc (II) acetylacetonate (0.3 mmol), iron (III) acetylacetonate (2.7 mmol), sodium oleate (2 mmol), and oleic acid (4 mL) were mixed with benzyl ether (20 mL) under a gentle flow of argon. The mixture solution was stirred by a magnetic stirring apparatus in an argon atmosphere and heated to 393 K for 1 h. The mixture was further heated to reflux temperature (≈573 K) under an argon blanket and maintained at the reflux temperature for 1 h. After cooling down to 300 K by removing the heat mantle, the NPs were collected by centrifugation. The size of the nanoparticles was regulated by a heating rate from 393 to 573 K. The morphology and size of the nanoparticles were characterized using a high-resolution (120 kV) transmission electron microscope (TEM, H750, Hitachi. Corp., Hitachi, Japan). PMMA bone cement was purchased from the Ningbo Hicron Biotechnology Limited Company (Ning Bo, China). Zn_0.3_Fe_2.7_O_4_ nanoparticles (200 mg) were dissolved in a methacrylic acid monomer (MMA, 10 mL), and 20 g PMMA powder was divided into 10 bottles of 2 g PMMA powder in a sterile environment. Two grams of PMMA powder and 1 mL of MMA containing Zn_0.3_Fe_2.7_O_4_ nanoparticles were mixed and injected into the bone marrow cavity of the tibia to fill the bone defect, and several minutes later, the mixture was solidified.

### 2.2. ICP-MS of PMMA-Zn_0.3_Fe_2.7_O_4_ Bone Cement

Three 200 mg solidified PMMA-Zn_0.3_Fe_2.7_O_4_ bone cement samples were prepared using the method mentioned above. The samples were dissolved by a mixture of nitrohydrochloric acid (HCL: HNO_3_ = 3:1), the iron in the mixture was measured by inductively coupled plasma mass spectrometry (ICP-MS), and the mass percent of nanoparticles in the bone cement was calculated using the chemical formula of Zn_0.3_Fe_2.7_O_4_.

### 2.3. Morphology Characterization

The SEM images were captured using a field emission electron microscope (SU8020, Hitachi. Corp., Hitachi, Japan) operating at 10 kV. The solidified PMMA-Zn_0.3_Fe_2.7_O_4_ bone cement samples were prepared and adhered to conducting resin. After metal spraying, surface characterization was captured by FE-SEM, and the element distribution was observed by energy disperse spectroscopy (EDS).

### 2.4. Mechanical Properties

The PMMA-Zn_0.3_Fe_2.7_O_4_ bone cement was prepared, and the bone cement pastes were injected into a cylindrical column mold at the dough stage and compacted with metal clips. Compressive strength test samples (12 mm in length and 6 mm in diameter) were prepared and dried for 24 h at room temperature. The bone cement’s pastes were poured into square molds at the dough stage and compacted by metal clips. Bending strength test samples (3.3 mm in thickness, 10 mm in width, and 75 mm in length) were prepared and dried for 24 h at room temperature. All compressive and bending strength samples were tested using an electronic universal testing machine (3380, Instron Corp., Norwood, MA, USA).

### 2.5. Cytotoxicity

Mouse embryonic fibroblast (MEF) cells were purchased from the American Type Culture Collection (ATTC, Rockville, MD, USA). Cells were all cultured in a DMEM/HIGH GLUCOSE (Hyclone, Logan, UT, USA) medium supplemented with 10% fetal bovine serum and antibiotics (100 µg/mL penicillin and 100 µg/mL streptomycin) in a humidified atmosphere of 5% CO_2_ at 37 °C. According to the guidelines of ISO5833, 2 g ball-shaped solidified PMMA-Zn_0.3_Fe_2.7_O_4_ bone cement was immersed in 1 mL of culture medium for 24 h in a humidified atmosphere of 5% CO_2_ at 37 °C to prepare the leaching solution. The PMMA leaching solution was prepared as described above. The two types of 100% leaching solutions were diluted to acquire a 50% leaching solution. The 50% and 100% PMMA-Zn_0.3_Fe_2.7_O_4_ and PMMA leaching solutions were incubated with MEF for 24 h, 48 h, and 72 h. The cell viabilities were evaluated by Cell Counting Kit-8 assay (CCK-8, Dojindo, Kyushu, Japan), and the absorbance was measured at 450 nm with a multimode plate reader (TECAN InfiniteM200 PRO, Switzerland). Cell viability was expressed as the percentage of viable cells compared with controls (cells incubated with pure culture medium).

### 2.6. Heating Efficiency

PMMA-Zn_0.3_Fe_2.7_O_4_ bone cement solidified samples (5 mm in diameter and 10 mm in length) were prepared. One hole (1 mm in diameter and 5 mm in depth) was drilled from the center of the sample basal circle. The PMMA samples were prepared using the same method described above. The cylinder-shaped samples were put into a 2.5 mL Eppendorf tube and covered with polystyrene foam to avoid thermal dissipation. A fiber optic probe (Neoptix Corp., CA, USA) was inserted into the previously drilled hole. The temperatures in the core of the samples were documented every second after switching on the AMF (HYPER5, MSI Corp., Flagstaff, AZ, USA) with a frequency of 430 kHz.

### 2.7. Preparation of the Rabbit Bone Metastases Model

New Zealand white rabbits were supplied by the laboratory animal center of the Chinese PLA General Hospital. The rabbits were approximately 20–24 weeks old and weighed 2.5–3.0 kg. All animal experiments were approved by the animal welfare ethics committee of the Chinese PLA General Hospital. The VX2 solid tumor mass was purchased from Tongpai Biological Technology Limited Company (Shanghai, China). After fast thawing in warm water (37 °C), the cryopreserved tumor mass was sheared into a small piece (1 mm^3^) by ophthalmic scissors, then collected by centrifugation and re-suspended by PBS. The VX2 tumor mass suspension was added to a 10 mL syringe.

After successful anesthetization with ketamine (10 mg/kg) and lumianning (5 mg/kg), the right hind leg skin of a rabbit was prepared, and the VX2 tumor mass suspension was injected into the muscles. Three weeks later, the tumor tissue of the tumor-bearing rabbit was harvested and sliced into small pieces to prepare the bone metastases model.

The rabbits were successfully anesthetized with ketamine (10 mg/kg) and lumianning (5 mg/kg), the right hind leg skin was prepared, and the puncture point was located 1 cm under the tibial medial plateau. After local anesthesia with 1% lidocaine, a 3 mm diameter puncture needle was penetrated into the tibia plateau, several fresh VX2 tumor masses (1 mm^3^) were put into the bone marrow cavity of the tibia through the bone hole, and the hole was sealed by bone wax. The rabbits were fed normally 6 h after the operation.

### 2.8. In Vivo Magnetic Hyperthermia Treatment of Bone Metastases

The bone metastases of rabbits were randomly divided into three groups of 10. Rabbits injected with M-PMMA and treated in AMF (HYPER5, MSI Corp., Flagstaff, AZ, USA) were included in the M-PMMA + AMF group; rabbits injected with M-PMMA without AMF, the M-PMMA group; and rabbits without M-PMMA and AMF, the control group. One week after the model establishment operation, all animal models were confirmed by X-ray examination (Optima XR220amx, GE, Boston, MA, USA). After the tumor-bearing rabbit was punctured vertically with a vertebroplasty puncture needle 3 cm into the tibial plateau of the affected limb, 1 mL of the contents of the bone marrow cavity was withdrawn to prevent spillage during the bone cement injection. Approximately 0.5–0.8 mL of magnetic bone cement was injected into the bone marrow cavity along the bone hole, and the M-PMMA injection was completed and treated in AMF (f = 430 kHz, H = 10–14 kA/m) for 30 min.

Magnetic hyperthermia began at 24 h post-M-PMMA injection. The rabbits in the M-PMMA + AMF group were administered magnetic hyperthermia treatment after one day, and all rabbits were treated seven times. Magnetothermal therapy uses medical tape to affix the optical fiber approximately 1 cm below the tibial plateau, and the affected limb is placed inside the magnetothermal therapy coil. The magnetic thermotherapy apparatus was turned on, and the magnetic field strength was adjusted to raise the temperature to 43 °C. The X-ray examination was carried out post-M-PMMA injection and at the end of magnetic hyperthermia. A chest CT scan (Optima TM CT670, GE, Boston, MA, USA) was also administered at the end of the magnetic hyperthermia. The weight and cross-section diameter of the tumor-bearing hind legs in each group were measured, and the appearance of the tumor-bearing hind legs was also observed. When the rabbits died, the tumor-bearing hind legs were harvested and scanned using micro-CT (Quantum, GX2, PerkinElmer, Norwalk, CT, USA); bone mass was measured using auxiliary software. The harvested tumor-bearing hind legs were decalcified and embedded in paraffin, the samples were sliced by microtome (2235, Leica, Wetzlar, Hessian, Germany) and stained by hematoxylin and eosin, and the pathological sections were observed by microscope (DMi1, Leica, Wetzlar, Hessian, Germany).

### 2.9. Statistical Analysis

The results are expressed as the mean ± standard deviation for as many continuous variables as possible. A normal distribution was confirmed, and significant differences were calculated using a one-way ANOVA. Non-normally distributed data were analyzed using the Kruskal–Wallis test, followed by the Mann–Whitney U-test to find significant differences between groups. Statistical analyses were performed using GraphPad Prism^®^ (7.04, GraphPad Software Inc., La Jolla, CA, USA). A *p* value of less than 0.05 was considered statistically significant.

## 3. Results

### 3.1. Morphology Characterization of PMMA-Zn_0.3_Fe_2.7_O_4_ Bone Cement

**Figure 1:**
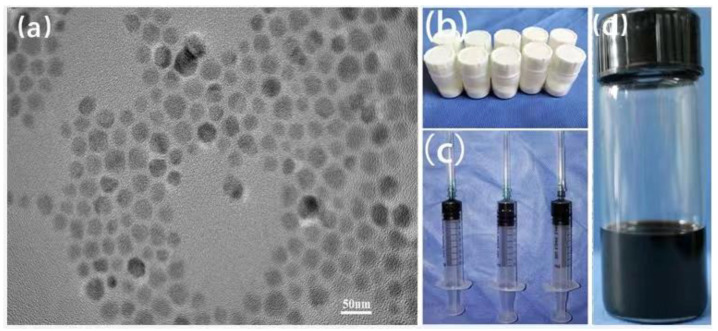
Morphology and characterization of Zn_0.3_Fe_2.7_O_4_ nanoparticles and PMMA-Zn_0.3_Fe_2.7_O_4_ bone cement. (**a**) TEM image of 22 nm Zn_0.3_Fe_2.7_O_4_ nanoparticles. (**b**) PMMA powder; each bottle contained 2 g of PMMA powder. (**c**) Methyl methacrylate (MMA) monomer contains Zn_0.3_Fe_2.7_O_4_ nanoparticles, with 20 mg Zn_0.3_Fe_2.7_O_4_ nanoparticles in 1 mL of MMA monomer. (**d**) Image of solidified magnetic bone cement.

The morphology characterization of Zn_0.3_Fe_2.7_O_4_ nanoparticles and PMMA-Zn_0.3_Fe_2.7_O_4_ bone cement is shown in Figure 1. The Zn_0.3_Fe_2.7_O_4_ nanoparticles mean size was 21.8 ± 2.0 nm and polyhedral in shape. TEM images (Figure 1a) show homogeneity in size and successful modification with no agglomeration.

The naked Zn_0.3_Fe_2.7_O_4_ nanoparticles are hydrophobic, so they can be well dispersed in the methyl methacrylate (MMA) monomer. The ratio of nanoparticles to the MMA monomer was 20 mg to 1 mL, and 2 g of PMMA powder (Figure 1b) was mixed and stirred with MMA with nanoparticles. The weight ratio of Zn_0.3_Fe_2.7_O_4_ nanoparticles incorporated into the PMMA bone cement matrix was 1%.

**Figure 2:**
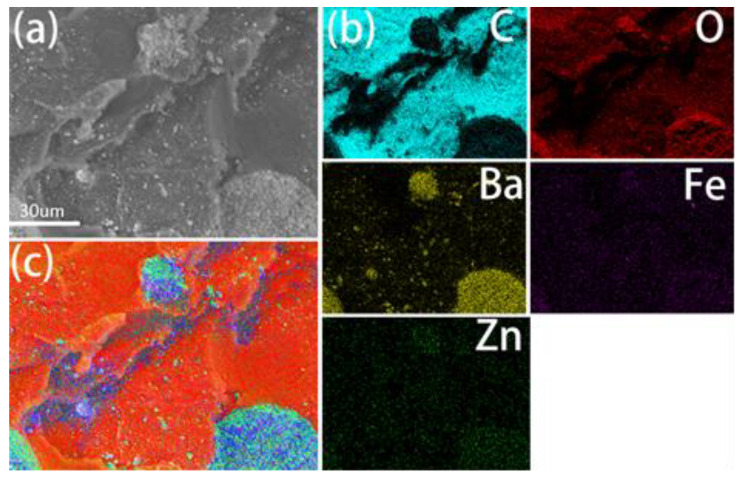
SEM image and element distribution (EDS-mapping) (**a**–**c**) of PMMA-Zn_0.3_Fe_2.7_O_4_ bone cement.

The solidified magnetic bone cement is shown in Figure 1c. The PMMA doped with 1 wt% Zn_0.3_Fe_2.7_O_4_ nanoparticles is black. The polymerized PMMA was densely distributed, and barium sulfate particles were embedded in the PMMA. Barium sulfate particles were doped as radiopaque agents, by which we were able to obtain clear and precise images in the process of injection. The results of the element distribution (EDS) mapping (Figure 2a–c) show that the Zn and Fe elements were uniformly distributed in the PMMA matrix.

### 3.2. Mechanical Properties and Cytotoxicity

**Figure 3:**
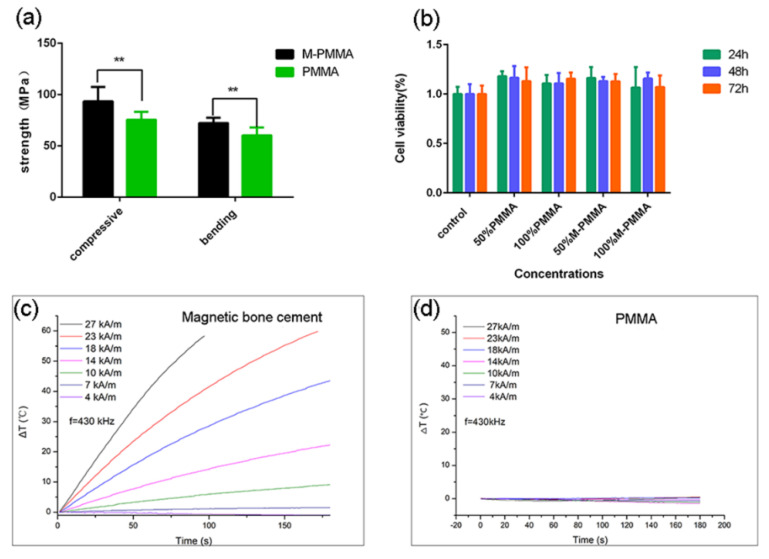
(**a**) Mechanical strength of PMMA-Zn_0.3_Fe_2.7_O_4_ bone cement comparable to PMMA. (**b**) Cytotoxicity of PMMA-Zn_0.3_Fe_2.7_O_4_ and PMMA bone cement leaching solution. (**c**,**d**) Heating curve of PMMA-Zn_0.3_Fe_2.7_O_4_ and PMMA bone cement in AMF (f = 430 kHz) (** *p* < 0.01).

Figure 3a shows the compressive and bending strengths of the M-PMMA and PMMA samples. The compressive strength of M-PMMA was 93.34 ± 6.29 MPa, and the bending strength was 72.18 ± 2.30 MPa, while the compressive and bending strengths of PMMA were 75.48 ± 3.45 MPa and 60.22 ± 3.39 MPa, respectively. There was a statistically significant difference between the two groups (*p* < 0.05). The mechanical strength of PMMA was enhanced after adding the Zn_0.3_Fe_2.7_O_4_ nanoparticles. The mechanical properties of M-PMMA meet the criteria listed in the ISO 5833:2002(E) guidelines, which require that the compressive and bending strength must be higher than 70 MPa and 50 MPa.

As shown in Figure 3b, the cell viability of the M-PMMA group was not significantly different from that of the other two control groups after 72 h of incubation (*p* > 0.05). Consequently, PMMA-Zn_0.3_Fe_2.7_O_4_ bone cement has no toxic effects on MEF cells, which means that it is biocompatible.

### 3.3. Heating Efficiency and Magnetic Heat Treatment Effects In Vivo

Figure 3c–d shows the heating curve of PMMA-Zn_0.3_Fe_2.7_O_4_ and pure PMMA in the AMF of 4–27 kA/m at a frequency of 430 kHz. As illustrated in Figure 3d, the surface temperature remained unchanged with time, even in high magnetic field strength (27 kA/m). This means that pure PMMA did not have the ability to generate heat. As shown in Figure 3c, although the surface temperature of PMMA-Zn_0.3_Fe_2.7_O_4_ barely increased in the low magnetic field strength (4–7 kA/m), when the field strength reached 10 kA/m, the temperature increased gradually. When the field strength was switched to 27 kA/m, the change in surface temperature increased to 60 °C within 100 s.

**Figure 4:**
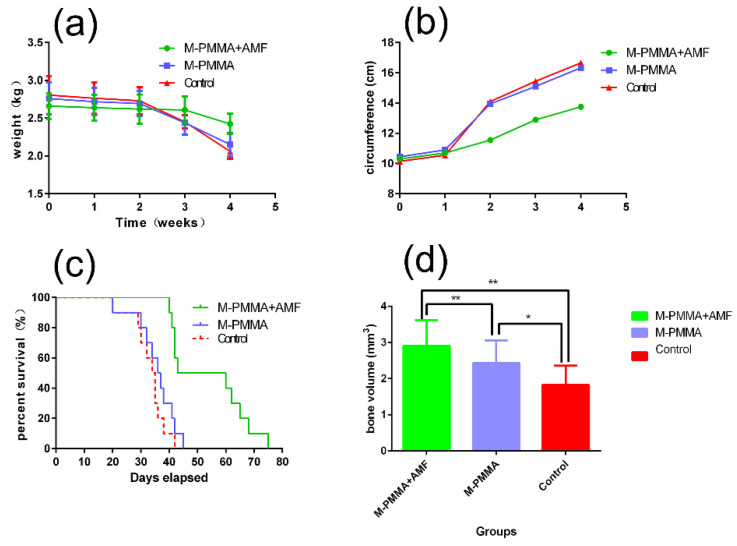
(**a**) Body weight changes within four weeks. (**b**) Tumor-bearing hind leg circumferences within four weeks. (**c**) Morbidity-free survival. (**d**) Bone volume of tumor-bearing hind legs (* *p* < 0.05, ** *p* < 0.01).

Figure 4a shows the body weight changes of rabbits in the three groups within four weeks after the establishment of the model. In the first two weeks, the weights of the three groups had no significant statistical difference (*p* > 0.05). One week after the model was established, the tumor-bearing hind legs began to swell. As shown in Figure 4b, the circumferences of the tumor-bearing hind legs in the three groups showed no significant difference in the first week (*p* > 0.05), but at the beginning of the second week, the difference became significant (*p* < 0.05). The condition of the rabbits worsened following lung metastases. Food intake decreased rapidly at three weeks post-model establishment in the M-PMMA and control groups, while such conditions appeared later in the PMMA + AMF group. Although all the tumor-bearing rabbits died in our experiment, the rabbits in the M-PMMA + AMF group lived longer. Figure 4c shows the morbidity-free survival curve of the three groups: the median survival time of the M-PMMA + AMF group was 51.5 days (40–75 days); the M-PMMA group was 36.5 days (20–45 days); and the control group was 34.5 days (20–42 days). A log-rank test showed that the survival time among the three groups had a significant difference (*p* < 0.05), and the survival time of the M-PMMA + AMF group was longer than the other two groups.

**Figure 5:**
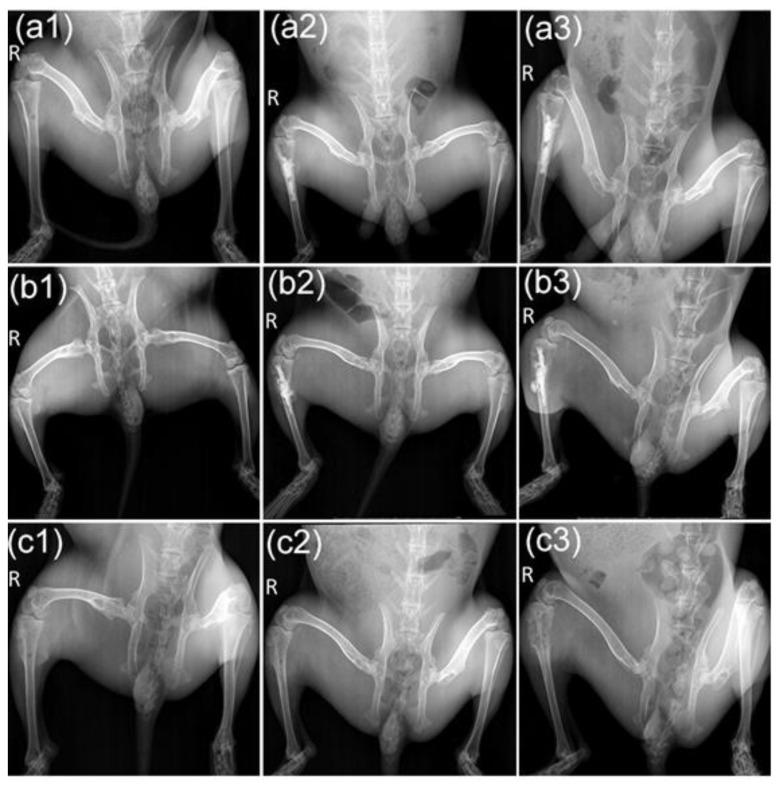
X-ray images of the animal models. Images of hind legs in the M-PMMA + AMF group pre-injection (**a1**), after (**a2**) magnetic bone cement injection, and at the end of magnetic hyperthermia (**a3**). Images of the M-PMMA group (**b1**–**b3**) and the control group (**c1**–**c3**) at the same time point.

We further evaluated bone resorption using X-ray and micro-CT scans. The X-ray image was first roughly examined using the morphologic changes of the tumor-bearing tibias. As shown in Figure 5, with the pre-injection of M-PMMA, the cortical bone thicknesses and trabecular separation of the tumor-bearing proximal tibias were similar to the healthy hind legs in the three groups (Figure 5a1,b1,c1). Post-injection of M-PMMA, high-density bone cement was located in the cavum medullare of the tumor-bearing proximal tibias (Figure 5a2,b2). The volume of M-PMMA was calculated before injection (0.7–1.0 mL). At the end of the magnetic treatment, the tumor-bearing proximal tibias of rabbits in the M-PMMA and control groups were severely destroyed, and the surrounding soft tissues were swollen (Figure 5c1,c2), while those in the M-PMMA + AMF group were less damaged (Figure 5c3). Although thinning of the cortical bone was observed, the tibial shape was well maintained in the M-PMMA + AMF group.

**Figure 6:**
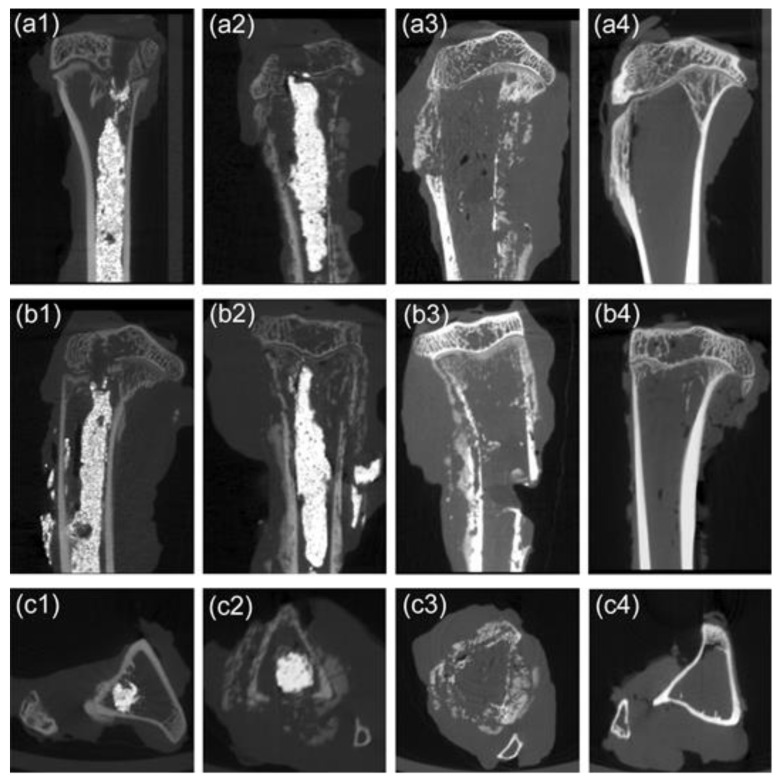
Micro-CT image of the affected lower extremities in the different groups after magnetic hyperthermia. Sagittal section (**a1**), coronal section (**b1**), and cross section (**c1**) of the M-PMMA + AMF group. Sagittal section (**a2**), coronal section (**b2**), and cross-section (**c2**) of the M-PMMA group. Sagittal section (**a3**), coronal section (**b3**), and cross-section (**c3**) of the control group. Sagittal section (**a4**), coronal section (**b4**), and cross-section (**c4**) of normal tibias.

In addition, the incidence of pathologic fractures was 10% in the M-PMMA and control groups, which did not occur in the M-PMMA + AMF group. Furthermore, skeletal morphologies and bone resorption were evaluated by micro-CT after magnetic hyperthermia. Although the cortical bone thickness of tumor-bearing tibias in the M-PMMA + AMF group (Figure 6a1,b1,c1) was less than in the normal tibias (Figure 6a4,b4,c4), skeletal morphology still existed. However, the tumor-bearing proximal tibias in the M-PMMA (Figure 6a2,b2,c2) and control groups (Figure 6a3,b3,c3) were severely destroyed, the cortical bone was erosive, and the skeletal morphologies were almost destroyed. The bone volume of the tumor-bearing proximal tibias was measured by software (Figure 4d), and the results were tested with one-way ANOVA. The bone volume in the M-PMMA + AMF group was higher than in the other two groups (*p* < 0.05). Magnetic hyperthermia induced by PMMA-Zn_0.3_Fe_2.7_O_4_ bone cement can inhibit bone resorption.

**Figure 7:**
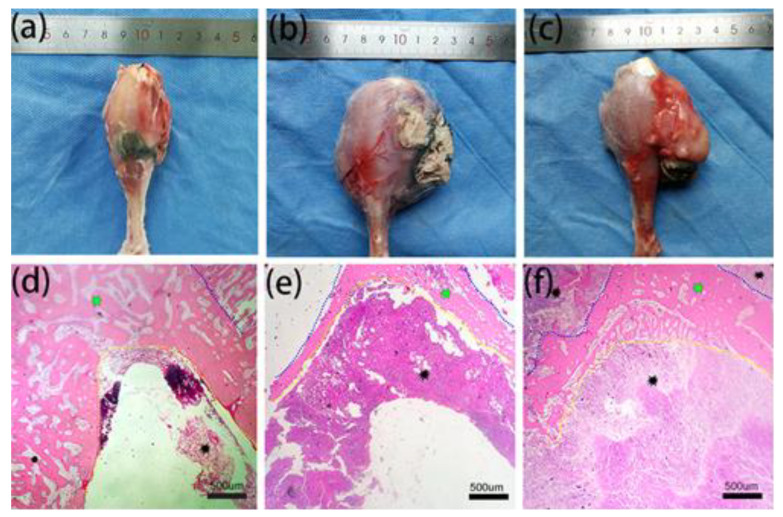
Sample photographs and hematoxylin and eosin staining of tumor-bearing tibias in the different groups. (**a**,**d**) show the M-PMMA + AMF group; (**b**,**e**), the M-PMMA group; and (**c**,**f**), the control group. The black asterisks in (**d**–**f**) are VX2 tumor cells, and the green asterisks are normal bone tissue. The bone tissue is the area between the yellow dotted line and the blue dotted line (hematoxylin and eosin stain, original magnification × 40).

Figure 7a–c shows the sample photographs of tumor-bearing hind legs in different groups. The circumference of the legs in the M-PMMA + AMF group (Figure 7a) was less than in the other two groups. In addition, the VX2 tumor cells invaded the surrounding muscles in the M-PMMA and control groups, and we found that the knee joints and condyles of the femurs of some rabbits were also damaged. Meanwhile, necrosis occurred in the M-PMMA (Figure 7b) and control groups (Figure 7c).

## 4. Discussion

We mixed 1 wt% Zn_0.3_Fe_2.7_O_4_ with good biocompatibility and high heating efficiency into a PMMA matrix to prepare M-PMMA, which minimized the affection for physicochemical properties and satisfied the hyperthermia requirement in the AMF. Magnetic bone cement is composed of magnetic nanoparticles and a bone cement matrix. The magnetic nanoparticles, the core of magnetic bone cement, directly determine the heating efficiency of AMF. We chose Zn_0.3_Fe_2.7_O_4_ nanoparticles as the threads in magnetic bone cement because of their high heating efficiency and good biocompatibility, which have been reported previously [12].

Zn_0.3_Fe_2.7_O_4_ nanoparticles were synthesized using a one-pot approach [13]. Generally, zinc-doped ferrite nanoparticles demonstrated soft magnetic performance, and the specific absorption ratio (SAR) was not very high, which was not the best choice for magnetic hyperthermia. However, we found that the saturation magnetization (Ms) of zinc-doped ferrite is very sensitive to Zn content, with ZnFeO_4_ being antiferromagnetic and Zn2+ ions occupying only the A-site of the spinal lattice. Consequently, Ms, and anisotropy can be tuned by changing the Zn:Fe ratio, and Ms can be increased by properly doping zinc elements in ferrite [12]. Zn_0.3_Fe_2.7_O_4_ nanoparticles have the highest SAR in a series of zinc-doped ferrite nanoparticles [10,11]. Previous studies have demonstrated that superparamagnetic iron oxide nanoparticles with an average size of 19 ± 3 nm had significant SAR values under clinical conditions [14]. Meanwhile, cube-shaped NPs lead to a large heat emission capability due to their minimized surface anisotropy, reduced spin disordering, and addition of exchange anisotropy [15].

The design and synthesis of magnetic bone cement to treat bone metastases has been reported [16,17]. However, the threads used in those experiments were iron powder or micron-dimensional ferrite nanoparticles at an early stage [18]. Due to the poor heating efficiency of these threads, investigators had to increase the weight percent in the magnetic bone cement to reach the clinical hyperthermia temperature (43 °C), sometimes by up to 40–60% [8,19]. Nevertheless, with this increase, the physicochemical properties of bone cement will be modified, including the mechanical strength, setting time, and polymerization temperature. Accordingly, the heating efficiency of the magnetic nanoparticles improves to decrease the weight percent, and the modification of the physicochemical properties of the bone cement matrix will be minimized.

Providing mechanical support and filling bone defects are the two basic functions of bone cement. Furthermore, mechanical support is especially important in the treatment of spinal osteolytic metastases because the spine supports the whole trunk. If the spine is unstable, axial pain and nerve symptoms will appear. In this work, the mechanical strength is improved by incorporating magnetic nanoparticles into the bone cement matrix, and the results are consistent with previous studies [16,20], whereby the mechanical strength improved with the increasing magnetic nanoparticle weight percent in the bone cement matrix. However, other researchers obtained contrary results: The mechanical strength was weakened after mixing the magnetic nanoparticles [8,21]. We believe that the mechanical strength may not only relate to the weight ratio of the magnetic nanoparticles but also to the mixture method. In other words, whether the magnetic nanoparticles are well distributed in the bone cement matrix may affect mechanical strength. If the weight ratio of the nanoparticles is high, they must be mixed with PMMA (or CPC) powder, but when the weight is low, the nanoparticles can be mixed with the MMA monomer (or water). The latter mixture method may make the nanoparticles well distributed in the liquid–solid phase transition, which may improve the mechanical strength. Figure 2b shows that the zinc and iron elements are well distributed in the PMMA matrix, so the mechanical strength in our study was improved.

Biocompatibility is essential for the biomedical application of bone cement [22]. Generally, the cytotoxicity of M-PMMA is related to the composition of the magnetic nanoparticles and PMMA. However, M-PMMA is a rapid liquid-solid phase transition material that can be injected into the bone metastases area at the drawing stage and will be solidified after several minutes. After solidification, the magnetic nanoparticles are permanently incorporated into the PMMA matrix, as PMMA is a non-degradable material. In our previous studies, we found that the biocompatibility of silica coated Zn_0.3_Fe_2.7_O_4_ nanoparticles (Zn_0.3_Fe_2.7_O_4_/SiO_2_) is better than naked Zn_0.3_Fe_2.7_O_4_ nanoparticles [12]. Due to the non-degradable and solidification features of PMMA, we simply used naked Zn_0.3_Fe_2.7_O_4_ nanoparticles as threads.

Hyperthermia occurs between 43 °C to 45 °C, while ablation temperatures are above 60 °C [23]. When the surface temperature of M-PMMA reaches 60 °C, the surrounding healthy tissues may also be simultaneously damaged, especially when bone cement leakage occurs. Therefore, the key problem is controlling the temperature increase rate rather than increasing the temperature rapidly. In addition, for the sake of the product, the magnetic field strength and frequency must be under 5 × 109 Am^−1^ s^−1^ [24], which is the safety limit of AMF. If the M-PMMA needs to be heated in a high-strength field, the magnetic field may harm the body itself. Hence, we chose Zn_0.3_Fe_2.7_O_4_ nanoparticles as threads due to their appropriate hyperthermia performance in a low-strength field.

Immunodeficient animal models are widely used in tumor research. Bone tumor cells are percutaneously injected into the flank of nude mice, and the model is established several days later. Although such bone tumor animal models are convenient to observe and measure [25], they may not be appropriate for magnetic bone cement research. Because magnetic bone cement was designed for bone tumor or metastasis treatment, the percutaneous bone tumor is more like a soft connective tissue tumor than a bone tumor. The biological characterizations are quite different from bone tumors. If these bone tumor models are applied in hyperthermia induced by magnetic bone cement, they may not imitate the function of bone cement in a clinical setting [26]. In addition, the leakage of bone cement increases. Although researchers injected bone tumor cells into the bone marrow cavity of nude mice tibias to establish a bone tumor model [27], the magnetic bone cement was difficult to inject into such a thin bone marrow cavity. The VX2 tumor is a squamous cell carcinoma induced by the human papillomavirus, which was first characterized by Shope and Hurst in 1933 [28]. New Zealand white rabbits are congenitally immunodeficient with VX2 tumors. The rabbit VX2 bone tumor model is similar to metastases and osteosarcomas, which are inclined to metastasize to the lungs early. Therefore, the VX2 tumor model is viewed as a suitable model to investigate bone tumors [29]. In this study, we used a 3-mm diameter bone puncture needle, which is used for percutaneous vertebroplasty in the clinic, to minimize injury to the rabbits and reduce the operation time. The operation time can be reduced to 20 min by this minimally invasive operation, and compared to the conventional procedure [18], we optimized the model establishment process. PMMA-Zn_0.3_Fe_2.7_O_4_ with high heating efficiency and good biocompatibility can be used for the magnetic hyperthermia treatment of bone metastases. In this work, the VX2 bone tumor was extremely similar to the bone metastases in the clinic. Most rabbits in the M-PMMA and control groups presented with lung metastases three weeks after model establishment. When the tumor cells metastasized to the lungs, the food intake of the rabbits decreased rapidly, and the body weight simultaneously decreased. Hence, an MRI scan was conducted before M-PMMA injection to observe whether the model establishment was successful and to determine the time of injection. Two weeks after model establishment, the tumor-bearing hind legs in the M-PMMA and control groups swelled rapidly, and areas of necrosis and cavitation occurred within the tumor. However, the tumor-bearing hind leg in the M-PMMA + AMF group swelled slowly. Accordingly, the corresponding time-weight and time-circumference curves revealed that magnetic hyperthermia can suppress bone metastases locally.

Bone metastasis patients will deteriorate if tumor cells metastasize to the visceral organs in the clinic [30]. Bone metastases can be divided into osteoblastic and osteolytic metastases [31]. Osteolytic metastases lead to bone resorption and pathologic fractures, which result in pain and dysfunction of the limbs. Furthermore, spinal osteolytic metastases may bring about spinal cord compression syndrome [30]. Therefore, inhibiting bone resorption is also important in bone metastasis treatment [27]. In our experiment, the X-ray results showed that the tumor in the proximal tibia of the M-PMMA group and the control group was severely destroyed, and the surrounding soft tissue was swollen. In the M-PMMA + AMF group, the injury was milder, and although the thickness of the cortical bone was reduced, the shape of the tibia was maintained. This was confirmed in the micro-CT results. Although the cortical thickness of the tumor-bearing tibia in the M-PMMA + AMF group was less than that of the normal tibia, bone morphology was still present, and bone volume at the upper end of the tibia was the largest. However, in the two remaining groups, the proximal tibia tumors were severely damaged, the cortical bone was eroded, and the bone morphology was almost completely destroyed. The microscopic therapeutic outcomes of tumor-bearing hind legs were evaluated by standard H and E staining. We chose the bone marrow cavity of the tibia as the region of interest. In the M-PMMA + AMF group, the tumor cells in the area of the bone marrow of the tibia were necrotic, the cortical bone was seldom damaged, and there were no tumor cells in the cortical bone or outside the tibia. However, in the M-PMMA (Figure 7b) and control groups (Figure 7c), the VX2 tumor cells invaded the whole cavum medulla of the tibia, the cortical bone was severely destroyed, and even the area outside the tibias was filled with tumor cells. Histopathological analysis explicitly indicated the local tumor suppression effect of PMMA-Zn_0.3_Fe_2.7_O_4_ bone cement exposed to AMF. All the rabbits died from visceral metastases in our experiment, which indicates that magnetic hyperthermia locally suppresses bone tumors and that bone metastases need multidisciplinary treatment. In addition, we found that the therapeutic effects were closely related to treatment time. Bone metastases are more difficult to inhibit if tumor cells invade the surrounding tissues. This result is consistent with oncology treatment guidelines, which require early detection and treatment [3,32]. We revealed that PMMA-Zn_0.3_Fe_2.7_O_4_-meditated magnetic hyperthermia could not only locally suppress bone tumors but also inhibit osteoclastic bone resorption.

## 5. Conclusions

In summary, we synthesized Zn_0.3_Fe_2.7_O_4_ nanoparticles with high heat efficiency and good biocompatibility. These nanoparticles were then incorporated with PMMA bone cement to develop a magnetic bone cement with 1 wt% Zn_0.3_Fe_2.7_O_4_. The PMMA-Zn_0.3_Fe_2.7_O_4_ bone cement not only provides reliable mechanical support but also high heat efficiency. In vitro studies have revealed the biocompatibility of the cement. In vivo investigations indicate that PMMA-Zn_0.3_Fe_2.7_O_4_-meditated magnetic hyperthermia can suppress bone metastases locally and inhibit osteolytic bone resorption. Our studies demonstrate that with heating emission ability improvement, the nanoparticles incorporated into the bone cement matrix will be further reduced, and the effect on physicochemical properties will be minimized. PMMA-Zn_0.3_Fe_2.7_O_4_ bone cement may be appropriate for clinical use.

## Data Availability

The data that support the plots within this paper and other findings of this study are available from the corresponding author upon reasonable request.
